# Roles of Exosomes in Cardiac Fibroblast Activation and Fibrosis

**DOI:** 10.3390/cells10112933

**Published:** 2021-10-28

**Authors:** Julia Hohn, Wenbin Tan, Amanda Carver, Hayden Barrett, Wayne Carver

**Affiliations:** Department of Cell Biology and Anatomy, University of South Carolina School of Medicine, Columbia, SC 29209, USA; julia.hohn@uscmed.sc.edu (J.H.); wenbin.tan@uscmed.sc.edu (W.T.); carveramandab@gmail.com (A.C.); hbarrett@wakehealth.edu (H.B.)

**Keywords:** fibrosis, fibroblast, heart, exosome

## Abstract

Alterations in the accumulation and composition of the extracellular matrix are part of the normal tissue repair process. During fibrosis, this process becomes dysregulated and excessive extracellular matrix alters the biomechanical properties and function of tissues involved. Historically fibrosis was thought to be progressive and irreversible; however, studies suggest that fibrosis is a dynamic process whose progression can be stopped and even reversed. This realization has led to an enhanced pursuit of therapeutic agents targeting fibrosis and extracellular matrix-producing cells. In many organs, fibroblasts are the primary cells that produce the extracellular matrix. In response to diverse mechanical and biochemical stimuli, these cells are activated or transdifferentiate into specialized cells termed myofibroblasts that have an enhanced capacity to produce extracellular matrix. It is clear that interactions between diverse cells of the heart are able to modulate fibroblast activation and fibrosis. Exosomes are a form of extracellular vesicle that play an important role in intercellular communication via the cargo that they deliver to target cells. While relatively recently discovered, exosomes have been demonstrated to play important positive and negative roles in the regulation of fibroblast activation and tissue fibrosis. These roles as well as efforts to engineer exosomes as therapeutic tools will be discussed.

## 1. Introduction

Fibrosis, or accumulation of excessive extracellular matrix and matricellular proteins, occurs in response to tissue damage or stress in most organs and is evident in many cardiovascular diseases [1,2]. Cardiac fibrosis contributes to altered myocardial structure, geometry and compliance and has been implicated as an independent predictor of mortality in patients with non-ischemic heart failure [3]. Despite the negative outcomes associated with fibrosis, little headway has been made in the development of therapeutic strategies directly targeting this condition. A number of hurdles have hampered the development of effective anti-fibrotic treatments including complex fibrotic signaling that involves many interacting pathways making therapeutic responses difficult to predict and resulting in undesirable side effects [4,5]. Understanding the regulation of fibrosis has become increasingly important as it has been appreciated that in some settings the fibrotic response is reversible, at least in its early stages [6]. This realization has generated renewed interest in the fibroblast and other ECM-producing cells as potential therapeutic targets.

Numerically, fibroblasts form the largest cell population in the heart and are classically defined as the primary cell type involved in the synthesis and degradation of the extracellular matrix, which provides structure for cardiomyocytes and other cardiac cells [7]. It is now clear that fibroblasts are quite heterogeneous and have diverse roles including a sentinel function within the myocardium and secretion of an array of paracrine factors that modulate myocardial physiology [8,9]. They also form an integrative network with other cells including cardiomyocytes that can impact cardiac electrical and mechanical function [10]. Hence, fibroblasts are multi-functional cells integral to cardiac development, homeostasis, pathogenesis, and repair. Fibrosis is typically accompanied by the appearance of cells termed myofibroblasts that have characteristics of fibroblasts including extracellular matrix production as well as contractile activity similar to smooth muscle cells [11]. In the heart, myofibroblasts are largely derived from the activation of resident fibroblasts, a process that is stimulated by diverse mechanical and chemical factors [12].

## 2. Intercellular Communication in the Heart

Intercellular communication is critical for development and homeostasis in multicellular organisms. This is particularly true in the heart which includes a number of resident cell types (myocytes, fibroblasts, endothelial cells, smooth muscle cells) and diverse transient cells (macrophages, lymphocytes, etc.). Communication between these cells can be accomplished via physical cell-to-cell interactions or via secreted factors, including factors (proteins, mRNAs, miRNAS and others) delivered via extracellular vesicles such as exosomes. Direct physical interactions between cells involve diverse structures including tight junctions, adherens junctions, desmosomes, and gap junctions. Of these, gap junctions are well-established mediators of direct cell-to-cell communication. In vertebrates, these are composed of proteins termed connexins, which form channels allowing regulated transfer of small molecules such as ions, peptides, and miRNAs between cells. Experimental deletion of connexin proteins in animal models results in developmental defects in multiple organs including skeletal muscle, blood vessels, bone, heart, and others [13,14]. Heart disease is accompanied by alterations in the organization of gap junctions and expression of connexins [15] and these proteins have emerged as potential therapeutic targets in several cardiovascular conditions including myocardial ischemia/reperfusion and arrhythmia [16]. Gap junctions are heavily localized to the intercalated discs of cardiomyocytes and have been traditionally viewed as the substrate for electrical coupling between these cells; however, it is now clear that connexins also mediate electrical coupling between myocytes and fibroblasts [10,17]. Studies have illustrated that reduced connexin expression precedes fibrosis in animal models of cardiovascular disease [18,19] and that genetic ablation of connexin 43 in cardiac fibroblasts promotes fibroblast activation and fibrosis [20]. While numerous questions remain regarding the roles of physical interactions of fibroblasts with each other and with other cells in myocardial fibrosis, this is an exciting area that may provide important clues regarding therapeutic modulation of this process. 

Many factors, including growth factors, hormones, and cytokines, are secreted directly into the extracellular space and mediate communication between cells typically via specific receptors and the activation of signaling pathways and gene expression programs. A number of secreted factors have been shown to stimulate myofibroblast formation and fibrosis including paracrine factors from cardiomyocytes and inflammatory cells and autocrine factors from myofibroblasts themselves. These secreted factors include cytokines such as interleukin-13 (IL-13) and transforming growth factor-beta (TGF-β), growth factors such as platelet-derived growth factor (PDGF), and hormones such as angiotensin II. Most of these factors are synthesized and released from cells via secretory pathways; however, it has become apparent that some of these factors and other “cargo” are transported via extracellular vesicles to regulate myofibroblast formation and fibrosis.

## 3. Extracellular Vesicles 

Extracellular vesicles are membrane-bound vesicles released into the extracellular space by most, if not all, cells. These vesicles carry lipids, proteins, nucleic acids, and metabolites that are derived from their cell-of-origin. Pioneering studies over seven decades ago illustrated the functional existence of platelet-derived extracellular materials in the blood clotting process that is removed following ultracentrifugation of blood [21]. Microscopic studies subsequently identified extracellular vesicles within the cartilaginous matrix of the tibial epiphyseal plate of mice and demonstrated the membrane-bound nature of these vesicles [22]. Seminal studies in the late twentieth century began to elucidate functional roles for extracellular vesicles in the immune response [23]. Research focusing on extracellular vesicles has grown substantially in the past two decades leading to an enhanced understanding of their diverse physiological and pathological functions and their potential as diagnostic and therapeutic tools.

The classification of extracellular vesicles is continually evolving but most schemes distinguish three major subtypes including exosomes, microvesicles, and apoptotic bodies [24]. Though overlap exists between these groups of extracellular vesicles, they are generally classified based on their size, content, biogenesis, and function. Exosomes generally range from 40 to 150 nm with biomarkers including TSG101 and tetraspanin proteins, microvesicles from 150 to 1000 nm, and apoptotic bodies greater than 1000 nm with biomarkers including matrix metalloprotease 2 and CK18 [25]. Exosomes are generally derived by the inward budding of endosomal membranes forming cytosolic multivesicular bodies whose fusion with the plasma membrane results in the release of exosomes. Direct blebbing of extracellular vesicles from the plasma membrane results in the release of larger multivesicular bodies of various sizes, i.e., ectosomes, while apoptotic bodies are released by cellular death. 

A number of functions have been postulated for extracellular vesicles; however, interest in these vesicles grew exponentially with the realization that they are able to mediate intercellular communication via delivery of their cargo to recipient cells. They can mediate communication locally within the same tissue or, since they are released into the bloodstream and other bodily fluids, between distant organs [26]. The composition of extracellular vesicles is altered with changes in the pathophysiological status of the cells of origin and these changes elicit distinct responses in the recipient cells. For example, the number and size of extracellular vesicles produced by vascular cells of individuals with port wine stain are increased [27] and these are thought to play important roles in the progression of this congenital disorder. Extracellular vesicles are found in all biological fluids including plasma, urine, and cerebrospinal fluid. Because extracellular vesicles retain proteins, lipids, and nucleic acids reflective of the pathophysiological status of their cells of origin, the diagnostic potential of these vesicles has also been recognized [28].

## 4. Exosomes 

Exosomes are a subset of extracellular vesicles that were originally thought to represent a mechanism for disposal of “cellular debris”. Mechanisms regarding their formation, secretion and uptake are beginning to be elucidated and will be briefly addressed here. These topics have been extensively discussed in recent reviews [29,30].

Biogenesis—Exosomes are produced via the endosomal system beginning with invagination of the plasma membrane to form early endosomes. Early endosomes fuse to form late endosomes with invagination of the endosomal membrane producing intraluminal vesicles. Late endosomes or multivesicular bodies can either fuse with lysosomes or autophagosomes to be degraded or fuse with the plasma membrane to release exosomes and their contents into the extracellular space.

While the molecular mechanisms regulating the formation and secretion of exosomes are far from understood, studies have indicated two general pathways, one dependent upon the endosomal sorting complex for transport proteins (ESCRT) and another that is ESCRT-independent [31]. Four different protein complexes comprise the ESCRT family, termed ESCRT-0, -I, -II and -III complexes. An additional accessory vacuolar protein sorting 4 (Vps4) complex has also been identified. Each of these complexes is comprised of multiple proteins. In the canonical ESCRT-dependent pathway, ESCRT complex proteins are recruited to the endosomal membrane sequentially [32]. Phosphatidylinositol-3-phosphate, abundant in endosomal membranes, initially recruits the ESCRT-0 complex to early endosomes. ESCRT-0 recruits ESCRT-I via interactions with TSG101 of the ESCRT-I family [33]. This results in the sequential recruitment of ESCRT-II and ESCRT-III to the endosome. ESCRT-III is required for the scission of intraluminal vesicles into the lumen of multivesicular bodies [34]. Additional non-canonical ESCRT-dependent pathways for intraluminal biogenesis and cargo sorting have also been identified including the ALIX-syndecan–syntenin pathway [35]. This pathway does not require ESCRT-0 but is dependent on ESCRT-III.

ESCRT-independent mechanisms of intraluminal vesicle formation have more recently been described through perturbation studies, which, despite eliminating ESCRT proteins, retained multivesicular body formation [31]. One such pathway involves the production of ceramide by neutral sphingomyelinase 2. Inhibition of ceramide production results in decreased exosome release in many cancerous cells while stimulation with C6 ceramide promotes exosome production [36]. 

Another potential ESCRT-independent pathway may be initiated by tetraspanins. The tetraspanins are a large family of integral cell surface proteins characterized by four transmembrane domains. These proteins are highly conserved and participate in a number of cellular processes including cell adhesion, differentiation, and tumor invasion [37,38]. Several of these proteins, including CD9, CD63 and CD81, are enriched in exosomes and are often used as biomarkers for this group of extracellular vesicles. They play roles in ESCRT-independent biogenesis, selective recruitment of biomolecules and secretion of exosomes as well as uptake of exosomes by target cells. CD9, CD63 and CD82 have been experimentally shown to impact exosome secretion [39]. Overexpression of CD9 has resulted in enhanced exosome secretion in several cell lines and CD63 ablation impaired intraluminal vesicle formation and exosome release independent of ESCRT or ceramide [40].

Trafficking of exosomes and secretion—As mentioned previously, multivesicular bodies can either be targeted to lysosomes for degradation or to the plasma membrane for release of exosomes. The mechanisms underlying the sorting of multivesicular bodies is beginning to be elucidated. In B-lymphocytes, two pools of multivesicular bodies have been identified based on cholesterol content [41]. Multivesicular bodies with higher cholesterol levels were targeted to the cell surface where they fused with the plasma membrane and released exosomes. ISGlyation, an ubiquitin-like modification, of multivesicular body proteins also appears to be involved in targeting these structures as ISGlyation promoted fusion with lysosomes instead of the plasma membrane [42].

Similar to other cytoplasmic vesicles, interactions of multivesicular bodies with the cytoskeleton, small GTPases and molecular motor proteins are essential for their transport to the cell surface. Increased or decreased expression of cortactin, an actin cytoskeletal regulatory protein, has resulted in enhanced or reduced extracellular vesicle secretion, respectively [43]. Cytoskeletal reorganization is essential for selective secretion of exosomes at the immunological synapse of B and T lymphocytes, a specialized structure at the interface between these cells and antigen-presenting cells. T lymphocyte activation leads to the trafficking of multivesicular bodies and the release of exosomes preferentially at the immunological synapse. While the molecular mechanisms of this process have not been entirely elucidated, studies have clearly demonstrated the essential role of cortical actin cytoskeletal reorganization in this process [44]. Furthermore, PKCδ appears to be important in actin reorganization at the immunological synapse and subsequent polarization of exosome secretion at this site [45]. The roles of members of the Rab family of small GTPases in vesicular transport and trafficking of exosomes to the plasma membrane have been well-described. In particular, functional screens identified fundamental but distinct roles for Rab27a and Rab27b in the docking of multivesicular bodies at the plasma membrane and exosome secretion [46].

Cells only release a very small proportion of their cellular contents via exosomes and the degree and rate of exosome secretion is dependent on the cell type and pathophysiological status of the cell. Tumor cells have been shown to release substantially more exosomes than non-tumorous cells [47]. Various types of cellular stress including hypoxia, oxidative stress and chemotherapeutic agents can modulate exosome secretion [48]. For instance, treatment of epithelial adenocarcinoma A549 cells with platinum nanoparticles has resulted in enhanced secretion of exosomes [49]. The fact that this response is inhibited by N-acetylcysteine indicates that oxidative stress induced by the platinum nanoparticles underlies the modulation of exosome secretion in this situation.

As mentioned above, fibroblast activation and fibrosis can be stimulated by mechanical forces. The effects of mechanical forces on exosome secretion are only recently beginning to receive attention. Studies in the cardiovascular system, where cells are continually exposed to changes in the mechanical environment, illustrated that experimentally induced increases in cardiovascular load via transaortic constriction elevated serum concentration of exosomes approximately 3-fold compared with control animals [50]. Treatment of isolated periodontal ligament cells with 20% uniaxial cyclic stretch also increased the secretion of exosomes [51]. Similar treatment of gum fibroblasts or dental pulp fibroblasts did not elicit an increase in exosome secretion, suggesting that the effects of mechanical forces on exosome secretion may be specific to particular cell types. Similarly, application of oscillatory strain to isolated triple negative breast cancer (TNBC) cells (4T1.2 or MDA-MB-231 cell lines) stimulated exosome secretion; however, similar forces had no effect on exosome secretion by estrogen receptor-positive MCF-7 cancer cells [52,53]. In these studies, exosomes from TNBC cells subjected to oscillatory strain were also more readily taken up by myeloid-derived suppressor cells and macrophages compared with exosomes from control cells. This suggests a mechanism whereby strain-induced exosome production by tumor cells may play a role in host immunosuppression. It will be very interesting to determine whether mechanical force-induced exosome production can affect fibrosis either directly via extracellular matrix-producing cells or indirectly by altering the inflammatory or immune responses.

Composition—Exosomes contain diverse proteins, lipids, and nucleic acids. The composition of exosomes is quite variable and is dependent in part on the cell type and pathophysiological status of the cell. Studies have even illustrated differences in content between exosomes released at the basolateral versus apical aspects of epithelial cells [54]. Recent studies have illustrated the utilization of different pathways for polarized exosomal release in epithelial cells [55]. In particular, the ALIX–syntenin 1–syndecan 1 system is essential for apical exosome release while sphingomyelinase-dependent ceramide machinery is required for basolateral release.

Particular miRNAs are enriched in exosomes relative to the whole cell, indicating that exosome content is regulated and that miRNAs can be targeted to the exosomes. Some mRNAs are also preferentially enriched in exosomes [56]. Multiple mechanisms have been shown to be involved in protein and RNA sorting into exosomes. In general, post-translational modifications such as phosphorylation, ubiquitination, glycosylation, myristoylation, and sumoylation can serve as sorting signals. RNA binding proteins or specific RNA motifs may also play functional roles in sorting or selective packaging of specific RNAs into exosomes. In addition, other levels of selectivity can be recruited to sort specific proteins into exosomes [25].

Exosome uptake by target cells—Once released into the extracellular environment, the ability of exosomes to impact physiological or pathological processes is typically dependent on uptake of the exosome and its cargo by target cells. The uptake of exosomes by recipient cells is far from understood but is thought to be mediated by multiple processes including phagocytosis, macropinocytosis, plasma membrane fusion and endocytosis via clathrin-dependent and independent mechanisms recently reviewed in [57]. Interestingly, recent studies have implicated a role for the extracellular matrix component fibronectin in uptake of extracellular vesicles by target cells [58]. Fibronectin has been shown to be an abundant component of extracellular vesicles produced by mouse and human hepatocytes. Ablation of fibronectin in mouse hepatocytes had no effect on extracellular vesicle biogenesis but resulted in significant reduction in the uptake of extracellular vesicles by hepatic stellate cells. Studies have illustrated that hepatocyte-derived extracellular vesicles can attenuate carbon tetrachloride-mediated liver fibrosis. Interestingly extracellular vesicles from fibronectin-ablated cells are equally as effective as those from normal cells in reducing carbon tetrachloride-induced fibrosis, despite alterations in uptake. 

## 5. Exosomes and Heart Disease

Understanding of the regulation of pathophysiological processes by exosomes in the heart is continuing to emerge, but it is clear that exosomes can have beneficial and detrimental effects on cardiac health [59]. Alterations in exosomal structure, quantity or composition could all theoretically impact cardiac homeostasis and contribute to pathogenesis [60,61]. Similar to other cells, essentially all cardiac cells secrete exosomes that contain a wide array of cargo, the composition of which is influenced by cellular phenotype [62,63]. Initial studies in this area have utilized cell culture models to illustrate that exosomes may provide a novel means of communication between diverse cell types within the heart and thereby modulate pathophysiological processes. Exosomes isolated from cultured neonatal cardiac fibroblasts rapidly entered cardiomyocytes in vitro, activated signaling pathways including Erk 1/2, p38 and Akt and induced cardiomyocyte hypertrophy [64]. Exosomes isolated from cardiac fibroblasts following myocardial infarction have also been demonstrated to induce hypertrophic gene expression in cultured cardiomyocytes [65]. On the other hand, exosomes containing miRNA-208a isolated from cardiomyocytes induced a fibrotic response when incorporated into fibroblasts [60]. These early experiments with isolated cells illustrating functional roles for exosomes have now been expanded to several animal models including myocardial infarction.

Preclinical and clinical studies have illustrated the effectiveness of stem cell therapy in improving outcomes following myocardial infarction [66]. Numerous studies have now demonstrated that the effectiveness of stem cell therapy in preventing or reversing myocardial damage is, at least in part, due to factors secreted by the stem cells. The cellular mechanisms through which these factors function to prevent deleterious myocardial remodeling are quite diverse and include attenuation of oxidative stress, prevention of cardiomyocyte apoptosis, stimulation of neovascularization and angiogenesis, enhancement of myocardial contractility, reduction of fibroblast activation/fibrosis and others. A number of studies have illustrated that miRNAs transported via stem cell exosomes are among the factors that modulate these processes. In vitro, exosomes isolated from cardiac progenitor cells prevented stress-induced apoptosis in HL1 and H9c2 cardiomyocyte lines [67]. Exosomes derived from mesenchymal stem cells have been shown to reduce infarct size, fibrosis, and cardiomyocyte apoptosis in rodent models of myocardial infarction [68]. These cardioprotective effects were at least partly due to specific miRNAs contained within the exosomes that modulated pro-survival and apoptotic pathways. Utilization of a combinatorial treatment approach, in which animals were treated with exosomes isolated from mesenchymal stem cells as well as mesenchymal cells themselves, following myocardial infarction yielded improved cardiac function and reduced infarct size compared with either treatment alone [69]. The beneficial effects found with this treatment appeared to be in part due to alterations in the microenvironment following exosome treatment that resulted in greater mesenchymal stem cell survival and retention in the injured myocardium. 

Circulating exosomes are being used as biomarkers of pathological conditions including cardiovascular disease (discussed below) and also impact cardiovascular function. Exosomes isolated from non-pathological animals delivered into the circulation of rats prior to induction of myocardial infarction diminished infarct size [70]. These exosomes also reduced the hypoxia-induced death of HL1 cardiomyocytes in vitro via an HSP70/TLR 4 pathway. Similarly, exosomes isolated from plasma of exercised rats (swimming protocol for four weeks) and injected into the myocardium prior to myocardial ischemia/reperfusion injury significantly reduced infarct size and improved myocardial function [71]. Gene expression and perturbation studies have illustrated that these protective effects were largely dependent on miRNA-342-5p contained in the exosomes from exercised animals. 

## 6. Role of Exosomes in Fibrosis and Fibroblast Activation 

**Figure 1:**
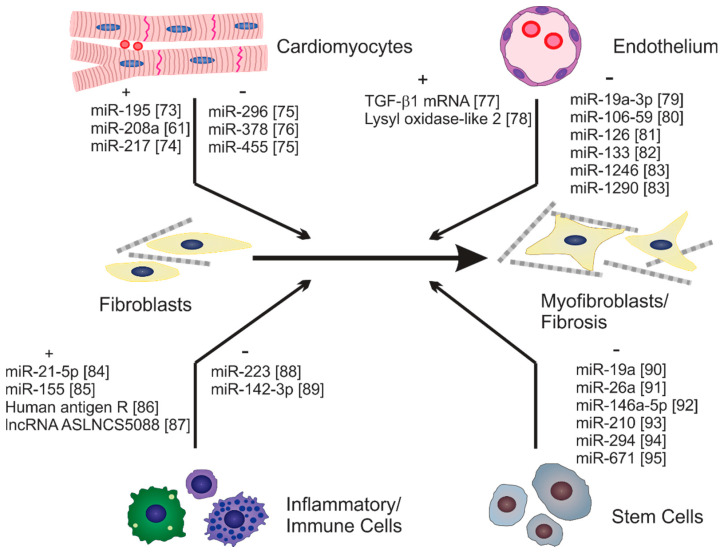
Cells that secrete exosomes modulating fibrosis. A schematic illustration of examples of identified cells and cargo that modulate fibroblast activation and/or fibrosis. Only components that have been functionally validated to alter fibroblast activation or fibrosis and whose cell-of-origin have been identified are included. Note that exosomes and their cargo can have both positive (+) and negative (−) effects on fibrosis [73,74,75,76,77,78,79,80,81,82,83,84,85,86,87,88,89,90,91,92,93,94,95].

Fibroblast activation and fibrosis can be induced by diverse mechanical and chemical stimuli. Recent studies have emerged indicating roles for exosomes in both negative and positive regulation of fibroblast activation and fibrosis, which is likely a reflection of the heterogeneity of exosomal cargo from diverse cells [65,72]. A variety of cells have been identified that secrete exosomes impacting fibroblast activation either directly or indirectly. Some of these and examples of their identified cargo are illustrated in Figure 1. 

Exosomes and miRNAs that promote fibroblast activation and fibrosis—As mentioned previously, exosomes carry a variety of cargo that is potentially involved in paracrine signaling between cells including proteins, lipids, mRNAs, and miRNAs. miRNAs (miRs, microRNAs) are highly conserved non-coding RNA molecules that are involved in the regulation of target gene expression. While they do not encode any protein, miRNAs regulate target gene expression post-transcriptionally and extensive research has focused on these as carriers of bioregulatory information in exosomes. A number of miRNAs have been shown to induce fibroblast activation and fibrosis in animal models or in vitro studies. The specific miRNAs involved appear to be numerous and their molecular mechanisms quite diverse [96,97]. 

Exosomal miRNAs in serum have been evaluated as potential biomarkers of diverse diseases including fibrosis and serum-derived exosomes can stimulate fibrosis. In one study, a panel of serum-derived exosomal miRNAs were able to distinguish patients with hepatic fibrosis resulting from chronic *Schistosoma* infection compared with healthy individuals [98]. One of these, miR-146a-5p, even distinguished among patients with differing grades of fibrosis. Recent studies have evaluated the potential functional role of serum-derived exosomal miRNAs in the pathological modulation of fibroblast phenotype and gene expression [61]. Exosomes isolated from serum of patients with congestive heart failure were enriched in miR-320a and levels of this miRNA correlated closely to functional and molecular biomarkers of heart failure progression. Transfection of the HEH2 myocardial fibroblast line with miR-320a mimics resulted in enhanced fibroblast proliferation, activation (α-smooth muscle actin expression) and collagen production. 

Exosome-mediated communication between resident fibroblasts and other resident myocardial cells can also be pro-fibrogenic. Several cardiomyocyte-derived exosomal miRs have been shown to promote fibroblast activation and fibrosis. miR-208a expression was elevated in cardiomyocytes in animal models of heart disease including myocardial infarction and doxorubicin-induced cardiotoxicity [61]. Expression of this miRNA was also increased in exosomes isolated from cardiomyocytes in vitro following treatment with angiotensin II or hypoxia. Incubation of cardiac fibroblasts with miR-208a-enriched exosomes promoted fibroblast proliferation, activation, and collagen production. Furthermore, treatment of mice with miR-208a antagomirs attenuated fibrosis and improved cardiac function following myocardial infarction. Another miRNA, miR-217, was one of several highly upregulated miRNAs from the hearts of patients with congestive heart failure [99] and was also increased in hearts of mice following banding of the thoracic aorta [100]. Treatment of isolated cardiomyocytes, but not cardiac fibroblasts, with angiotensin II has resulted in increased miR-217 expression and increased secretion of miR-217 in exosomes [74]. Incubation of cardiac fibroblasts with exosomes from cardiomyocytes treated with angiotensin II resulted in enhanced fibroblast proliferation and expression of extracellular matrix components. While many questions remain regarding the roles of exosomes in communication between resident cells of the myocardium, these studies clearly indicate that fibroblasts can take up exosomes generated by other myocardial cells, and that these can modulate fibroblast phenotype and gene expression.

**Figure 2:**
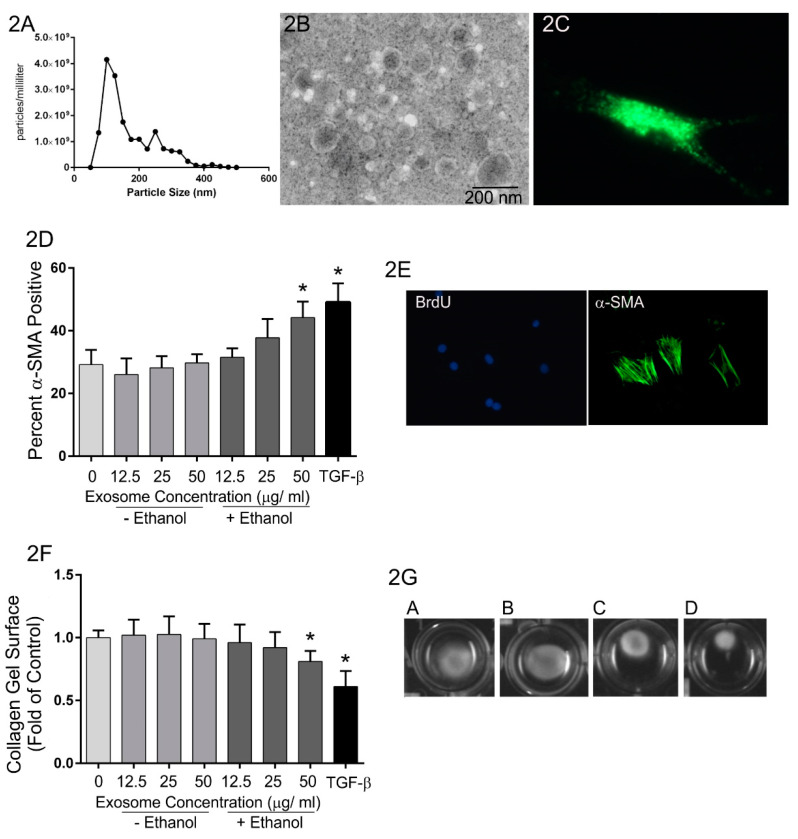
Effects of H9c2 cardiomyocyte-derived exosomes on fibroblast activation. H9c2 cells were treated with 0 or 200 mg/dL ethanol for 24 h, exosomes purified from the conditioned medium and used to treat cardiac fibroblasts. (**A**,**B**) Purified exosomes were analyzed by NanoSight and transmission electron microscopy illustrating that the majority of the vesicles were less than 200 nm in diameter. (**C**) Purified exosomes were labeled with fluorescent PKH67 and used to treat cultured cardiac fibroblasts. Representative fluorescent microscopy image illustrating abundant PKH67-labeled exosomes associated with a cardiac fibroblast in vitro. (**D**) Quantification of α-smooth muscle actin-positive fibroblasts following treatment with no exosomes (negative control) or varying doses of exosomes isolated from H9c2 cells treated with 0 or 200 mg/dL ethanol. Following purification, the protein concentration of exosomes was determined using the bicinchoninic acid assay (Pierce BCA Protein Assay). The concentrations of exosomes used for treatment of fibroblasts was standardized based upon total protein concentration. TGF-β1 treatment (5 ng/mL) was included as a positive control. (**E**) Images of control cells illustrating BrdU-positive nuclei used to count total cells and anti-α-smooth muscle actin immunocytochemistry used as a marker of myofibroblast formation. (**F**) Quantification of collagen hydrogel contraction as an indirect bioassay of fibroblast activation following treatment with no exosomes (negative control) or varying doses of exosomes isolated from H9c2 cells treated with 0 or 200 mg/dL ethanol. TGF-β1 treatment (5 ng/mL) was included as a positive control. (**G**) Representative collagen gels following treatment for 24 h with: (**A**) no exosomes, (**B**) 50 μg/mL exosomes from H9c2 cells not exposed to ethanol, (**C**) 50 μg/mL exosomes from H9c2 cells treated with 200 mg/dL ethanol and (**D**) TGF-β. (* ≤ 0.05 compared with untreated controls based upon One way ANOVA, *n* = 4 independent experiments including independent fibroblast cultures and independent isolations of exosomes).

Along these lines, previously unpublished studies from our group have been carried out to evaluate the role of exosome-mediated communication between heart myocytes and fibroblasts in the pro-fibrotic response to alcohol abuse. Chronic abuse of alcohol deleteriously impacts most organ systems and approximately one-third of individuals who abuse alcohol long-term develop alcoholic cardiomyopathy. This condition is accompanied by myocardial remodeling that includes cardiomyocyte apoptosis and fibrosis and often results in heart failure [101]. In these experiments, H9c2 cardiomyocytes were treated with or without ethanol (200 mg/dL) for 24 h and exosomes purified from conditioned medium by filtration followed by ultracentrifugation. Extracellular vesicles generated by this purification scheme were somewhat heterogeneous in size with most below 100 nm in diameter (Figure 2A,B). Isolated rat cardiac fibroblasts were then treated with exosomes and the expression of α-smooth muscle actin and the contraction of three-dimensional collagen gels evaluated as assays of fibroblast activation (Figure 2D–G). Exosomes isolated from H9c2 cells not exposed to ethanol had no effect on the expression of α-smooth muscle actin (Figure 2D) nor on contraction of three-dimensional collagen hydrogels by fibroblasts (Figure 2F,G). In contrast, treatment of fibroblasts with exosomes isolated from H9c2 cells exposed to ethanol demonstrated increased α-smooth muscle actin staining and enhanced collagen hydrogel contraction. While the molecular mechanisms of this effect have not been identified, these data suggest that exposure to ethanol likely induces alterations in cardiomyocyte exosomal cargo, which in turn promotes fibroblast activation. 

miRNAs derived from cardiomyocyte exosomes not only act on fibroblasts directly but can modulate their activity indirectly by acting on other cells that impact fibroblast activity and fibrosis. For instance, several studies have shown that cardiomyocyte-derived exosomes can modulate inflammatory cell behavior and phenotype [102,103]. miR-23a is widely involved in diverse pathophysiological processes and has been shown to promote myocardial hypertrophy [104]. In response to angiotensin II, atrial myocytes secreted exosomes enriched in miR-23a [105]. miR-23-enriched exosomes prevented macrophage polarization to an M2 phenotype and, via factors secreted by the macrophages, promoted atrial fibrosis. 

In addition to secreted biochemical factors, exosome-mediated communication between inflammatory/immune cells and fibroblasts may be involved in cardiac fibrosis. Expansion and activation of CD4+ T cells facilitates myocardial remodeling; however, the molecular mechanisms of this are not entirely known [106]. Treatment of isolated cardiac fibroblasts with exosomes from activated CD4+ T cells resulted in enhanced fibroblast proliferation, activation, and pro-fibrotic gene expression. Furthermore, treatment of mice with activated CD4+ T cell exosomes exaggerated the effects of myocardial infarction including diminished myocardial function and further expansion of infarct size. These effects were shown to be at least partly dependent on miR-142-3p transported in the activated CD4+ T cell exosomes. Inflammatory cell-derived exosomal miRNAs are also important mediators of fibrosis in non-cardiac models. In an experimental model of silicosis, macrophages exposed to silica particles secreted exosomes with almost three hundred differentially regulated miRNAs compared with non-silica treated macrophages [107]. Among these differentially expressed miRNAs, miR-125a-5p was increased and shown to down-regulate Smurf1, an E3 ubiquitin-protein ligase and inhibitor of BMP signaling. Uptake of miR-125a-5p containing exosomes resulted in activation of cultured fibroblasts likely as a result of increased Smad signaling. 

Exosomes and miRNAs that inhibit fibroblast activation and fibrosis—A number of exosomal-derived miRNAs have been identified that inhibit fibroblast activation and fibrosis and are being proposed as potential therapeutic candidates. In particular, exosomes derived from diverse stem cells appear to be protective against myocardial damage and fibrosis. For instance, treatment of rats with exosomes derived from mesenchymal stem cells was shown to prevent cardiac inflammation and fibrosis and improve cardiac function following myocardial infarction even better than treatment with stem cells themselves [108]. In vitro studies suggested the cellular mechanisms of this effect was in part due to prevention of cardiomyocyte apoptosis and fibroblast activation and that this was likely due to miRNAs transported in the stem cell exosomes. Similar studies illustrated that treatment of mice with exosomes derived from bone marrow mesenchymal stem cells preserved myocardial function and inhibited cardiomyocyte apoptosis following infarction [109]. These effects were mediated by the targeting of Bax by exosome-derived miR-150-5p. Other studies have illustrated that treatment of mice with exosomes from adipose-derived mesenchymal stem cells also protected against myocardial infarction-induced damage via miR-671, which has been demonstrated to modulate TGF-β signaling [95,110].

Exosomes from non-stem cells including inflammatory cells have also been shown to protect against myocardial damage. During cardiac injury, activated macrophages secrete exosomes that are enriched in miR-155 [85]. Fibroblasts readily incorporated macrophage-derived exosomes in vitro resulting in decreased fibroblast proliferation and increased production of inflammatory cytokines. Myocardial infarction of mice with genetically ablated miR-155 resulted in increased numbers of activated fibroblasts (α-smooth muscle actin-positive) and enhanced fibrotic scar formation compared with animals with intact miR-155. 

miR-29 has been studied extensively as inhibitory to fibrosis of the heart and other organs and has a unique role in modulating extracellular matrix expression directly. The miR-29 family contains three members, miR-29a, miR-29b and miR-29c, encoded by two gene clusters. miR-29 family members directly target at least sixteen extracellular matrix components including several collagen isoforms, elastin and fibrillin [111]. miR-29 expression was downregulated following myocardial infarction and after treatment of isolated heart fibroblasts with TGF-β [112]. Overexpression of miR-29 inhibited the pro-fibrotic response in isolated cardiac fibroblasts and in animal models [113,114]. These studies focused on endogenous miR-29 in cardiac fibroblasts; however, recent studies have shown that miR-29 carried by exosomes is able to reduce skeletal muscle atrophy and kidney fibrosis in a unilateral ureteral obstruction-induced model of kidney disease [114]. The role of exosomally-derived miR-29 in the modulation of cardiac fibrosis remains to be investigated. 

Non-miRNA Cargo and Fibrosis—Exosomes also have the ability to modulate the extracellular matrix via other mediators in addition to miRNAs. While these have not been as extensively studied as miRNAs, there are examples of their roles in fibrosis of the heart and other organs. Other types of RNAs can be transported by exosomes and impact fibrosis either directly or indirectly through modulation of the inflammatory response. Following kidney injury, exosome production is increased by renal tubule epithelial cells [115]. Exosomes isolated from hypoxic kidney tubule epithelial cells are able to activate fibroblasts and promote a fibrotic response. These exosomes were shown to contain TGF-β1 mRNA and treatment of kidney tubule epithelial cells with TGF-β1 siRNA diminished the response of fibroblasts to the epithelial cell-derived exosomes. Long noncoding RNAs (lncRNAs) contained in exosomes also modulate fibroblast activation and fibrosis. Recent studies have illustrated that exosomes from TGF-β-treated M2 macrophages contain abundant lncRNA-ASLNCS5088 that can be efficiently transferred to fibroblasts and elicits fibroblast activation in vitro [87]. The mechanisms of this response are at least partly due to the inactivation of miRNA-200c-3p by this lncRNA. Other studies have shown that exosomal lncRNAs also play an important role in myocardial infarction by mediating communication between cardiomyocytes and cardiac fibroblasts. Recently, it has been discovered that treatment of cardiac fibroblasts with exosomes derived from hypoxic cardiomyocytes inhibited fibroblast proliferation, migration, and invasion while promoting apoptosis [116]. RNA-seq analyses revealed that lncRNA139128 was overexpressed in hypoxic cardiomyocytes and cardiomyocyte-secreting exosomes. In vivo studies demonstrated that exosomal AK139128 exacerbated fibroblast apoptosis in myocardial infarcted rats. Several proteins transported by exosomes have been demonstrated to modulate fibrosis including the RNA-binding protein human antigen R [86], glycolysis-related proteins [117], Wnt proteins [118] and others. Exosome production is increased in several models of kidney disease and exosomes produced by renal tubule epithelial cells have been demonstrated to induce renal interstitial fibroblast activation [119]. Exosomes derived from renal tubule epithelial cells stimulated with TGF-β1 are rich in sonic hedgehog (Shh) and knockdown of this morphogen impairs the ability of the exosomes to induce fibroblast activation. 

Proteins associated with exosomes not only modulate fibroblast behavior but may also remodel the extracellular matrix directly. Alterations in the protease to anti-protease balance, particularly neutrophil alpha1 antitrypsin, results in extracellular matrix degradation in the lungs and chronic obstructive pulmonary disease (COPD). Recent studies have illustrated that exosomes from activated polymorphonuclear leukocytes (PMNs) may contribute to COPD and other respiratory diseases associated with extracellular matrix degradation [120]. Increased levels of matrix-degrading proteases were found to be associated with exosomes from activated PMNs. In particular, neutrophil elastase was shown to associate with the surface of exosomes from activated PMNs, which appears to make this protease resistant to cleavage by alpha1 antitrypsin. These exosomes were able to bind to and degrade type I collagen fibers via Mac1 integrin and neutrophil elastase, respectively. Furthermore, delivery of exosomes from activated PMNs to naive mice resulted in the appearance of symptoms of COPD including airway resistance, alveolar enlargement, and right ventricular hypertrophy.

As mentioned above, the cargo carried by exosomes that modulate fibrosis and fibroblast activation, their cells of origin and their mechanisms of action are quite diverse. The specific exosomal cargo that have been functionally demonstrated to affect cardiac fibrosis are summarized in Table 1.

## 7. Therapeutic Utilization and Engineering of Exosomes

Due to difficulties in delivering fragile therapeutics to the pathological myocardium, aggressive attempts are being made to identify novel delivery methods including nanomaterials, hydrogels, and others. Exosomes possess a number of advantageous properties that have led to their exploration as potential carriers of therapeutic cargo [134]. These include their small size and membrane properties that allow them to cross biological membranes including the blood-brain barrier and their intrinsic ability to target specific cells. The enclosure of their cargo within a lipid bilayer also helps protect exosomal components and reduces immunogenicity. Beyond these inherent properties, approaches are being developed to further engineer exosomes to enhance their therapeutic utility including modulation of their cargo, bioavailability, and delivery.

An important consideration for optimizing exosomes as a delivery vehicle is modulating their content to treat particular pathological conditions. A hurdle in this regard is loading cargo across the exosomal membrane. Several approaches have been developed to enhance the loading of specific molecules into exosomes via cargo pre-loading or post-loading strategies. The first approach is to engineer the donor cells producing the exosomes to express the component(s) of interest, which is then incorporated into exosomes as they form. Various vectors that express proteins or miRNAs of interest have been utilized to engineer exosome-producing cells and exosomes then harvested for downstream applications. This approach has been taken in a number of studies to load exosomes with specific miRNAs identified to treat particular pathological conditions. For instance, after miRNA-124a was identified as a potential treatment for glioblastoma, mesenchymal stem cells were engineered to highly express this miRNA via lentiviral infection [135]. Exosomes from these cells produced high levels of miRNA-124a, reduced the viability of glioblastoma cells in vitro and enhanced survival of mice with glioblastoma. Similar studies have recently been carried out engineering bone marrow-derived mesenchymal stem cells to express a miR-338 mimic via Lipofectamine transfection [136]. Treatment of H9c2 cardiomyocytes with miR-338 mimic-containing exosomes reduced apoptosis following exposure to hydrogen peroxide. Furthermore, delivery of these exosomes in a rat myocardial infarction model improved cardiac function even better than non-engineered exosomes. Similarly, engineering adipose-derived stem cells to express miR-146 and subsequent treatment of rats with exosomes produced by these cells reduced myocardial infarction-induced inflammation and fibrosis. The molecular mechanisms of this response involved inhibition of early growth response factor 1 expression and attenuation of toll-like receptor 4/NF-κB signaling. 

The cargo post-loading approach has been used to load specific cargo, such as therapeutic drugs or compounds, directly into purified exosomes. Loading may be accomplished via passive mechanisms in the case of hydrophobic compounds that diffuse through the lipid bilayer of the exosome or active mechanisms including electroporation or sonication of the exosomes. Passive loading efficiency varies greatly depending on the properties of the cargo and incubation parameters (duration, temperature, and other conditions). An advantage of exosomes for drug delivery is their ability to transport multiple classes of cargo simultaneously. Recent studies have engineered exosomes as a potential approach to more effectively treat cancer cells resistant to the chemotherapeutic compound 5-fluorouracil (5-FU). Purified exosomes were loaded with an inhibitor to miRNA-21 and 5-FU by electroporation [137] and demonstrated a significant reduction in 5-FU resistance and inhibition of cancer growth in a mouse model compared with either treatment alone.

Delivery of exosomes into the circulation often leads to accumulation primarily in the liver and spleen with much less accumulation in the heart or other organs. Approaches are being developed to improve cell-specific targeting of exosomes largely through incorporation of molecules into the exosomal membrane. Advances in this area would allow systemic delivery while reducing off-target effects and potentially lowering the effective dose of exosomes required for treatment. Similar to engineering the cargo delivered by exosomes, modulating the exosome surface could be done via engineering the donor cells or the exosomes themselves. The approaches for therapeutic exosome targeting include incorporation of surface targeting peptides, antibodies, receptor proteins or signaling molecules [138]. Several peptides have been incorporated into the exosome surface to target these to the heart. Stem cells engineered to incorporate one of these, termed ischemic myocardium targeting peptide, into the exosome membrane showed enhanced uptake into H9c2 cardiomyocytes compared with non-engineered exosomes. Treatment of mice with these exosomes following myocardial infarction resulted in increased accumulation of exosomes in the infarcted region, as well as reduced inflammation, apoptosis, and fibrosis. 

An innovative approach has been used to attract exosomes from the circulation to damaged myocardium following myocardial infarction using magnetic nanoparticles [139]. The investigators engineered nanoparticles composed of an Fe_3_O_4_ core and a silica shell. The outer layer of these nanoparticles contained antibodies that recognized CD63 of exosomes or myosin light chain surface markers of injured cardiomyocytes. In response to a magnetic field, nanoparticles were recruited to the injured myocardium and exosomes released. This resulted in reduced infarct size and improved left ventricular function.

## 8. Diagnostic Value of Exosomes

The current gold standard for assessment of many diseases including fibrosis involves invasive procedures such as tissue biopsies that often include significant patient risks. Development of biomarker profiles of disease that can be obtained from biological fluids including serum are being aggressively pursued. The properties of exosomes including relatively stable cargo that are reflective of pathophysiological status of tissues makes these ideal candidates for assessment of pathogenesis. The utilization of exosome-derived biomarkers diagnostically is most advanced in the cancer field where exosomal miRNA signatures have been developed for rectal, cervical, and other cancers [140,141] and the utilization of exosomal biomarkers has been commercialized [142].

With regards to cardiovascular disease, exosomal miRNA patterns are being established for several conditions [143] and may provide biomarkers on par with or better than current protein markers. Myocardial injury leads to the rapid appearance of cardiomyocyte-specific proteins, including cardiac troponins, in the bloodstream, which are widely used as biomarkers for acute myocardial infarction. Cardiomyocytes produce multiple miRNAs that are abundantly expressed by these cells and are increased in the bloodstream following myocardial injury [144]. Studies have indicated that some of these miRNAs are detected earlier in the bloodstream following myocardial injury and are more sensitive for myocardial damage than troponins [145,146]. In addition, unlike protein biomarkers, some of these miRNAs that are transported in exosomes have been detected in urine following myocardial injury [147]. These markers may also provide important information regarding disease progression and severity. For instance, an increased level of miR-208a has been demonstrated in exosomes isolated from individuals with coronary artery disease [148] and lower exosomal miR-208a levels were correlated to decreased mortality. Exosomal miRNA profiles are also being used to distinguish between cardiovascular diseases [149]. Next generation sequencing of miRNAs from serum-derived exosomes identified a panel of miRNAs that distinguished samples from acute myocardial infarction and cardiac sarcoidosis patients and both of these from control samples. 

As mentioned previously, a number of miRNAs have been identified that are associated with fibrosis. Analysis of exosomes from patients with idiopathic pulmonary fibrosis has demonstrated an increase in miRNAs indicative of fibrosis (specifically miR-7 and miR-125) and a decrease in anti-fibrotic miR-141 [150]. In a separate study, miR-21-5p has been correlated to bleomycin-induced lung damage and fibrosis in a mouse model [151]. The association of exosomal miR-21-5p with disease progression was demonstrated in patients with idiopathic pulmonary fibrosis and was found to be a predictor of mortality risk in these patients. Similar studies have been carried out in patients with kidney fibrosis. Interstitial fibrosis and tubular atrophy are major causes of allograft dysfunction following kidney transplantation. The ability to efficiently determine the degree of kidney fibrosis would be very beneficial following kidney transplantation. Recent studies have illustrated that the levels of miR-21 in exosomes isolated from patient plasma is a better indicator of the severity of interstitial fibrosis than currently used protein markers or other profibrotic exosomes [152]. 

Technology is also being advanced to more effectively isolate and detect exosomal biomarkers. Exosomes are recovered from biological fluids as a mixture with other components and require purification via methods that preferably provide high throughput, low contamination, and high recovery rate. Approaches that have been commonly utilized to purify exosomes include ultracentrifugation, ultrafiltration, size-exclusion chromatography, and immunological separation. Each has limitations including costly equipment, lack of sensitivity, low specificity and others. In recent years, microfluidics-based techniques have emerged as innovative approaches for exosome purification and detection [153]. Due to their precise nanoscale liquid and particle control, microfluidic devices have the potential for rapid and high yield purification of exosomes [154]. Research is aggressively taking place to advance strategies for microfluidic purification and detection of exosomes. 

The detection of miRNAs from biological fluids for diagnostic purposes is typically via polymerase chain reaction (PCR) or next-generation sequencing. Limitations in the clinical applicability of these including sensitivity of standard PCR and cost of specialized equipment are driving the search for alternative methods. A recent study presented a novel electrochemical biosensor-based approach to detect exosomal miR-181 in serum samples from healthy individuals and patients with coronary heart disease [155]. This approach was highly specific for miR-181, very sensitive with detection in the fM range and clearly distinguished control and diseased samples.

## 9. Conclusions

Fibrosis is a common consequence of tissue damage and is associated with a number of cardiovascular diseases including myocardial infarction, hypertension, aortic stenosis, atherosclerosis and others. Functional roles for exosomes and their cargo are being identified in fibroblast activation and fibrosis in the heart and other organs. These include both beneficial and detrimental effects likely due to specific cargo transported by exosomes under particular pathophysiological conditions. The reflection of exosomal cargo on the pathophysiological status of cells and tissues are making these valuable diagnostic biomarkers, and profiles are being established for multiple pathological processes. The size and structure of exosomes provide substantial benefits in the delivery and protection of cargo. These properties are being leveraged to engineer exosomes as systems for the delivery of therapeutic reagents.

## Figures and Tables

**Table 1:** Exosomal cargo that modulate cardiac fibrosis. Cargo that has been identified to modulate cardiac fibrosis or activation of cardiac fibroblasts is presented along with its cell(s) of origin, pathophysiological effects, and molecular mechanisms (if known). Only cargo whose functions have been experimentally verified are included.

Exosomal Cargo	Exosome Source	Pathophysiological Effects	Molecular Mechanisms	Reference
miR-19a-3p	Endothelial cells	Enhanced cardiac function and angiogenesis and reduced myocardial fibrosis in a mouse MI model.	Downregulation of thrombospondin 1 and increased expression of VEGFR2	[79]
miR-21	Stem cells	Preserved cardiac function and reduced apoptosis and fibrosis in rat MI model.	Modulation of PTEN-Akt pathway.	[121]
miR-21-5p	Mesenchymal stem cells	Improved cardiac function and reduced infarct size in mouse MI model. Enhanced macrophage polarization to the M2 phenotype. Reduced H9c2 apoptosis in response to oxygen glucose deprivation.	Reduced expression of pro-apoptotic genes.	[68,122]
miR-21-5p	Cardiac telocytes	Improved cardiac function, reduced infarct size and fibrosis and enhanced angiogenesis in a rat MI model. Promoted cardiac microvascular endothelial cell survival and tube formation in vitro.	Silencing of Cdip1 and downregulation of Caspase-3	[123]
miR-21-5p	Macrophages	Further reduction of cardiac function, increased cardiomyocyte apoptosis and expansion of fibrosis in mouse MI model.	Downregulation of TIMP 3	[124]
miR-22	Mesenchymal stem cells	Improve cardiac function and reduce myocardial apoptosis and fibrosis in a mouse MI model.	Suppression of Mecp2 expression	[125]
miR-30e	Bone marrow-derived mesenchymal stem cells	Reduced infarct size and myocardial fibrosis in rat MI model. Attenuated oxygen glucose deprivation-induced H9c2 apoptosis.	Reduction in expression of LOX1 and NF-kB p65/Caspase-9 signaling.	[126]
miR-126	Adipose-derived mesenchymal stem cells Umbilical cord blood-derived cells	Reduced infarct size and fibrosis in rat model of MI. Improved cardiac function, reduced myocardial hypertrophy and fibrosis in mouse model of diabetes (db/db).	Silencing of miR-126 target genes including Spred-1, VCAM and MCP1	[127,128]
miR-142-3p	CD4+ T cells	Aggravated cardiac dysfunction, infarct size and fibrosis in mouse MI model. Enhanced fibroblast proliferation, activation and pro-fibrotic gene expression in isolated fibroblasts.	Enhanced Wnt signaling via downregulation of Adenomatous Polyposis Coli (APC) expression	[106]
miR-146	Adipose-derived stem cells	Reduced inflammation and fibrosis in MI model.	Inhibition of early growth response factor 1 expression and attenuation of TLR4/NF-kB signaling.	[129]
miR-146a-5p	Cardiosphere-derived cells	Improved cardiac function and reduced myocardial fibrosis in porcine dilated cardiomyopathy model.	Unknown	[92]
miR-150-5p	Bone marrow-derived stem cells	Preserves cardiac function and inhibits cardiomyocyte apoptosis in MI model.	Downregulation of Bax.	[109]
miR-208a	Cardiomyocytes	Promotes fibroblast activation and fibrosis.	Decreased expression of Dyrk2.	[61]
miR-210	Hypoxia-treated mesenchymal stem cells	Improved cardiac function and reduced myocardial fibrosis in a mouse MI model. Enhanced tube formation by endothelial cells and reduced cardiomyocyte apoptosis in vitro.	Unknown	[93]
miR-217	Cardiomyocytes	Enhanced fibroblast proliferation and cardiac hypertrophy.	Modulation of PTEN/Akt pathway.	[74]
miR-218-5p miR-363-3p	Endothelial progenitor cells	Enhanced fibroblast mesenchymal to endothelial transition and angiogenesis in vitro. Improved cardiac function and reduced fibrosis in rat MI model.	Regulation of p53 and junction-mediating regulatory protein (JMY) expression	[130]
miR-290–295 cluster	Embryonic stem cells	Enhanced cardiomyocyte survival and neovascularization and reduced fibrosis in a mouse MI model.	Unknown	[94]
miR-320a	Serum	Promoted fibroblast activation and proliferation.	Regulation of PIK3CA/Akt/mTOR signaling.	[97]
miR-338	Bone marrow-derived mesenchymal stem cells	Improved cardiac function and inhibited cardiomyocyte apoptosis in rat MI model and in H9c2 cells.	Reduced MAP3K2 and JNK expression.	[131]
miR-671	Adipose-derived stem cells	Reduced cardiomyocyte apoptosis, inflammation and fibrosis in MI model.	Inactivation of TGFBR2/Smad2 axis.	[110]
miR-1246 miR-1290	Endothelial progenitor cells	Improved cardiac function and reduced infarct size and fibrosis in rat MI model. Enhanced fibroblast mesenchymal to endothelial conversion and angiogenesis in vitro.	Enhanced expression of ELF5, SP1 and CD31 in cardiac fibroblasts.	[83]
lncRNA ZFAS1	Human cardiac myocytes	Exaggerated decrease in cardiac function and increase in myocardial fibrosis in chronic kidney disease mouse model. Promoted fibroblast activation and pro-fibrotic gene expression in isolated fibroblasts.	Modulation of Wnt4/β-catenin signaling via miR-4711-5p regulation.	[132]
Human antigen R (HuR)	Macrophages	Increased fibrosis in angiotensin II infusion mouse model. Enhanced pro-inflammatory and pro-fibrotic gene expression in isolated fibroblasts.	Unknown	[86]
Decreased HSP70	Serum of aged rats	Stimulation of fibroblast activation, proliferation and pro-fibrotic gene expression in vitro.	Unknown	[133]

## Data Availability

Raw data are available from the authors upon request.
